# Longitudinal and age trends of metabolic syndrome and its risk factors: The Family Heart Study

**DOI:** 10.1186/1743-7075-3-41

**Published:** 2006-12-05

**Authors:** Aldi T Kraja, Ingrid B Borecki, Kari North, Weihong Tang, Richard H Myers, Paul N Hopkins, Donna Arnett, Jonathan Corbett, Avril Adelman, Michael A Province

**Affiliations:** 1 From the Division of Statistical Genomics, Washington University School of Medicine, Saint Louis, MO, USA; 2 Department of Epidemiology, University of North Carolina, Chapel Hill, NC, USA; 3 Division of Epidemiology and Community Health, University of Minnesota, Minneapolis, MN, USA; 4 Department of Neurology, Boston University Medical Center, MA, USA; 5 Department of Internal Medicine, University of Utah Health Sciences Center, Salt Lake City, UT, USA; 6 Department of Epidemiology, University of Alabama School of Public Health, Birmingham, AL, USA

## Abstract

**Background:**

We report longitudinal changes in the metabolic syndrome (MetS) in 2,458 participants from 480 families in the Family Heart Study. Participants were examined between 1994–96 (FHS-T1) and 2002–03 (FHS-T2), about 7.4 years apart. Additionally, the impact of medication on estimates of MetS prevalence, and associations of MetS with prevalent coronary heart disease (CHD) and type 2 diabetes (T2D) were studied.

**Methods:**

Three definitions for MetS prevalence were considered. One represented the original (**o**) National Cholesterol Education Program (NCEP) MetS criteria. Two others considered the confounding of medications effects, respectively (**m**) lipid medications constituted a categorical diagnostic criterion for lipids variables, and (**c**) lipids and blood pressure variables were corrected with average clinical trials medications effects. Logistic regression of MetS on CHD and T2D, as well as the trend analysis of MetS by age, were performed.

**Results:**

MetS increased from 17.1% in FHS-T1(**o**) to 28.8% in FHS-T2(**o**); from 19.7% in FHS-T1(**m**) to 42.5% in FHS-T2(**m**); and from 18.4% in FHS-T1(**c**) to 33.6% in FHS-T2(**c**). While we observed adverse changes in all risk factors, the greatest increase was for waist circumference (25%). The percentages of MetS were about 2 to almost 3 times higher in ages 50 years and older than in younger ages. The odds of having prevalent CHD were about 2.5 times higher in the subjects classified with MetS than without.

**Conclusion:**

MetS percentages increased noticeably longitudinally and cross-sectionally with older age. These conclusions were reached with and without considering medication use, but correcting risk factors for medications use affects the MetS prevalence estimates. As found in other studies, MetS was associated with increased odds for prevalent CHD.

## Background

The metabolic syndrome (MetS) is a combination of interconnected risk factors for obesity, insulin resistance and glucose intolerance, dyslipidemia, and hypertension. The Adult Treatment Panel III of the National Cholesterol Education Program (NCEP) defined a group of five clinical criteria for effective classification of MetS (see Methods) [1]. An individual that meets three or more of these criteria yields a clinical diagnosis of MetS.

Based on the NCEP classification, it is reported, that a rapidly growing epidemic of metabolic syndrome is taking place in the United States [2-4]. For example, Ford and Giles reported that about 24% of US adults (n = 8,608) from 20–70 years of age from the Third National Health and Nutrition Examination Survey (1988–1994), a cross-sectional health survey of a nationally representative sample of the US civilian population, were affected by MetS [2]. Meigs, summarizing data for the epidemiology of MetS and diabetes, stated that more than 8% of the US population from 20–74 years of age were affected by diabetes mellitus, which is often associated with MetS [3]. The prevalence of MetS varies with age and was 6.7% in subjects 20–29 years old and about 40% in those aged 60 years or more [3]. In a study (n = 3,510) of the US population, the prevalence of MetS was about 44% in participants 50 years and older [4].

MetS is of special interest because it represents a complex disorder expressed by interrelated risk factors with unknown genetic and not well known environmental influences. Previous familial studies have shown that MetS has an important heritable component [5-8]. While the genetic aspects of MetS are quite important, several studies have revealed that lifestyle modifications and/or medication use reduce the prevalence of MetS [1,9].

One of the main features of MetS is an increased risk of CHD and Type 2 diabetes (TD2) [10-12]. Alexander et al. reported that participants who had MetS but not diabetes had a 13.9% prevalence of CHD, whereas those with MetS and diabetes had a 19.2% prevalence of CHD [4]. Only a few studies have documented longitudinal effects of MetS, in its association with CHD and T2D [13-15]. We studied the same sampled population in the Family Heart Study (**FHS**) – Time 1 (FHS-T1) and FHS – Time 2 (FHS-T2), which represent two clinical visits with a mean age interval of about 7.4 years. The main purpose of our study was to assess the longitudinal trends of MetS, its association with age, prevalent CHD and T2D, and the impact of medication on the prevalence estimates of MetS.

## Sampled populations and methods

### Participants

The sampled population is part of the multi-center FHS supported by the National Heart, Lung, and Blood Institute. It is important to mention that the original FHS white familial sample was separated into two large groups, one random and one non-random, based on the participants' recruitment criteria for CHD and their familial risk for CHD. Details are provided elsewhere [[16], and [10-12]]. There were originally 5,718 participants in the FHS-1 (1994–96), but only large families were re-examined (2002–03). In our study, the FHS-T1 and FHS-T2 samples embodied the same 2,458 participants, from 480 families with a maximum of up to 16 members, with at least one measurement on the 5 MetS risk factors analyzed at both clinical visits. For a specific trait measured, if a subject had only FHS-T1 measured and FHS-T2 was missing, or vice versa, that measure was set to missing. Blood pressure (BP) and glucose (GLUC) variables were handled with special care. If systolic (SBP) and diastolic blood pressure (DBP) were missing in FHS-T1, the SBP and DBP in FHS-T2 were set to missing. Also, the anti-hypertensive medication use variable was set to missing. Similarly, if GLUC of FHS-T1 was missing, GLUC of FHS-T2 was set to missing (vice versa for Time 2 to Time 1), as well as the diabetes state and diabetes medication use variables. Our goal was to restrict the data to individuals with measurements at both time points for each risk factor. Such a sample, with two time clinical visits and a mean age difference of about 7.4 years, was the focus of our longitudinal comparisons.

### NCEP metabolic syndrome definition

The percentages of MetS were identified by applying the NCEP ATP III metabolic syndrome criteria (Table 1) [1]. An individual was classified as having MetS if they had a combination of any three or more of the following risk factors beyond their thresholds: waist circumference (WAIST) > 102 cm in men and > 88 cm in women; triglyceride (TG) levels ≥ 150 mg/dl; high-density lipoprotein cholesterol (HDLC) levels < 40 mg/dl in men and < 50 mg/dl in women; SBP OR DBP ≥ 130/85 mm Hg OR use of anti-hypertensive medications; and GLUC ≥ 110 mg/dl OR use of anti-diabetic medications. We label this **o**riginal MetS definition as (**o**). The second method, labeled (**m**), considered **m**edication use for TG and HDLC as a categorical effect and anyone on lipid lowering drugs was classified as positive for the NCEP lipid threshold (Table 1). This second method (**m**) is similar to the recent criteria proposed by the Scientific Statement of AHA/NHLBI for diagnosing and managing MetS, with a difference that they defined the threshold of fasting glucose to be 100 mg/dl [17]. To increase the precision of the MetS prevalence estimation, we also considered a third method labeled (**c**), where the original measurements of SBP, DBP, TG, and HDLC were **c**orrected with the average effect observed in clinical trials (Table 1) [[18], and unpublished data]. For anti-hypertensive medication(s), the participant's SBP and DBP were corrected by adding 14.8 and 10.5 mm Hg respectively [18]. The average effects employed for these corrections were a summary of 137 anti-hypertensive mono-drug therapy clinical trials with a total of 10,405 participants, including trials of 6 medication classes [18]. TG and HDLC of a participant using anti-hyperlipidemic medication(s) were similarly corrected by dividing respectively the measured values with by the following coefficients (1–15.2/100) and (1+6.1/100). The 15.2 and 6.1 represent the average percentage effects of anti-hyperlipidemic medications on TG and HDLC, respectively. The correction coefficients used here were estimated from 28 clinical trials with 18,742 participants and 2 medication classes as monodrug therapy [unpublished data]. The +/- sign in front of the average percentage effect (+6.1/-15.2) shows the effect's direction (increasing/decreasing) assuming that the medication altered the corresponding individual's risk variable.

**Table 1:** Diagnostic Criteria for Metabolic Syndrome Utilized in this Study

		(o) Method*	(m) Method**	(c) Method***
**No**	**Five NCEP criteria†**	**Categories**	**Categories**	**Categories**
1	Elevated WAIST	> 102 cm in men > 88 cm in women	Idem¶ Idem	Idem Idem
2	Elevated Fasting GLUC	≥ 110 mg/dl OR use of anti-diabetic medications‡	Idem Idem	Idem Idem
3	Elevated TG	≥ 150 mg/dl	Idem OR antihyperlipidemic treatment‡	If treated with antihyperlipidemics, the original TG was evaluated as TG/(1–15.2/100)‡ ≥ 150 mg/dl
4	Reduced HDLC	< 40 mg/dl in men < 50 mg/dl in women	Idem Idem OR antihyperlipidemic treatment‡	If treated with antihyperlipidemics, the original HDLC was evaluated as HDLC/(1+6.1/100)‡ < 40 mg/dl in men < 50 mg/dl in women
5	Elevated Blood Pressure	≥ 130 mm Hg SBP OR ≥ 85 mm Hg DBP OR medication treatment for hypertension‡	Idem Idem Idem	If treated with antihypertensives, the original SBP was evaluated as SBP+14.8 mm Hg‡ AND the original DBP was evaluated as DBP+10.5 mm Hg‡ ≥ 130 mm Hg SBP OR ≥ 85 mm Hg DBP

*Original NCEP MetS criteria; **Subjects treated with antihyperlipidemics were considered as passed the corresponding threshold criterion; ***Average effects of many clinical trials were used to impute the original values of treated TG, HDLC, SBP, and DBP. After this correction, categories of MetS were applied. † Any three of the five criteria constituted a diagnosis of MetS; ‡See Methods for details; ¶Identical with the left column category of the same row.

### Coronary heart disease

Prevalent CHD was based on self report and/or hospital validation of myocardial infarction, coronary bypass surgery, or coronary angioplasty. In the FHS-Time 1, CHD events were validated with hospital records. In the FHS-T2, the CHD events were self reported.

### Type 2 diabetes

T2D was defined by a fasting GLUC ≥ 126 mg/dl or current use of hypoglycemic medication(s) that was documented as diabetes self-reported history at the examination in the clinic. An age of onset ≥ 40 years was also required to diagnose T2D [19].

### Anthropometric, biochemical and derived measurements

All anthropometric measurements, including weight (kg), height (m), and waist circumference (measured at the level of the umbilicus in cm), were taken in all recruitment centers by trained technicians. Trained interviewers obtained the protocol information based upon questionnaires about the status of hypertension, medication use, coronary heart disease risk factors, and many more. All measured variables or subjects' responses collected were in accordance with FHS standard procedures. Sitting blood pressure measurements for FHS-T1 were collected with random zero mercury sphygmomanometers by certified technicians. Those for FHS-T2 were measured using Dinamap 1846-SX [20]. The detectable SBP ranges were 30–245 mm Hg and for DBP were 10–210 mm Hg. In both studies, the SBP and DBP were measured three times after the subject was asked to sit for five minutes. The mean of the second and third measures for systolic and diastolic represent the derived measures used in this study.

Venipuncture blood samples were drawn and collected at each field center. Blood biochemistries were quantified at the Central Biochemistry Laboratory at Fairview-University Medical Center in Minneapolis, MN. The variables of fasting GLUC (where fasting time was defined as ≥ 12 hours before blood draw) measured in mg/dl, HDLC measured in mg/dl and TG measured in mg/dl represent some of the blood biochemical assays. Details of each procedure regarding blood collection and blood assays are provided elsewhere [11].

### Statistical analysis

Identification of MetS groups was performed using programs written in the SAS language v. 9.1.3 for Linux OS (SAS Institute, Cary, NC). Procedure *FREQ *and *LOGISTIC *regression were carried out to analyze the associations of MetS with CHD and T2D, and to obtain the respective odds ratios. The Cochran-Armitage trend test was applied in SAS to test trends of MetS in decades of age groups.

## Results

The mean age difference of subjects between the FHS-T1 and FHS-T2 exams was about 7.4 years (Table 2). WAIST increased from a mean of 96.7 cm in FHS-T1 to 99.3 cm in FHS-T2. Similar increases were noted for GLUC and SBP. SBP and DBP at FHS-T2 showed a notable increase, especially after a correction for anti-hypertensive medications use. On the contrary, HDLC values decreased across exams, as well as after corrections for anti-hyperlipidemic medications use. The mean of TG decreased from FHS-T1(**o**) to FHS-T2(**o**), but it increased from FHS-T1(**c**) to FHS-T2(**c**) (Table 2).

**Table 2:** Participants' characteristics

**Variables**	**N**	**Mean**	**Std. Dev.**	**Mean**	**Std. Dev.**	**Mean**	**Std. Dev.**	**Mean**	**Std. Dev.**
		**Original FHS-Time1**		**Original FHS-Time2**		Corrected FHS-Time1*		**Corrected FHS-Time2**	

**AGE**	2458	50.6	13.0	58.0	13.0	-	-	-	-
**BMI**	2337	27.6	5.3	29.0	5.6	-	-	-	-
**WAIST**	2334	96.7	14.9	99.3	16.0	-	-	-	-
**GLUC**	1877	97.1	23.1	100.0	21.4	-	-	-	-
**HDLC**	2417	50.1	14.5	48.8	14.3	50.0	14.5	48.1	14.3
**TG**	2419	148.9	100.3	144.1	92.1	149.2	100.9	151.2	97.8
**SBP**	2330	116.0	17.0	121.2	20.3	118.9	20.0	126.4	23.6
**DBP**	2330	69.0	9.7	69.7	9.8	71.1	11.3	73.4	11.4
**Ratio smokers**									
**vs. non-smokers**	-	0.41	-	0.26	-	-	-	-	-
**Ratio males**									
**vs. females**	-	0.82	-	0.82	-	-	-	-	-

*The value of a trait was corrected if a participant used medication for improving the profile of lipids/of BP (see Methods)

**Figure 1:**
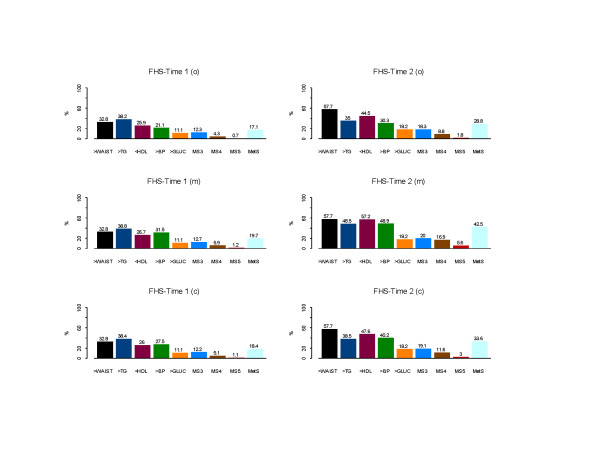
Percentages of MetS and its risk factors in the FHS-Time 1 and FHS-Time 2. Three analyses were applied: FHS-T1 (**o**)/FHS-T2 (**o**) – **o**riginal MetS (no medication effects on lipids were considered); FHS-T1 (**m**)/FHS-T2 (**m**) – **m**edication effects on BP, lipids, and GLUC were considered as categorical effects; FHS-T1 (**c**)/FHS-T2 (**c**) – for participants that used anti-hypertensive/anti-hyperlipidemic medication(s), **c**orrections of the corresponding risk factors with clinical trials medication average effects for BP and lipids were performed (see Methods). **Footnote**. MS3, MS4, MS5 are the percentages of participants with at least 3, 4, and 5 risk factors beyond the MetS NCEP thresholds.

Estimates of MetS percentages were 17.1% for FHS-T1(**o**) and 28.8% for FHS-T2(**o**) when medication use for lipids was not considered. The additional categorical correction of risk factor levels for lipids medication use produced the highest MetS percentages: 19.7% for FHS-T1(**m**) and 42.5% for FHS-T2(**m**), while the third method of correction of the lipid and blood pressure values yielded prevalence estimates of 18.4% and 33.6% at baseline and follow-up. The percentage change of MetS from FHS-T1 (**o**), (**m**), and (**c**) to FHS-T2 (**o**), (**m**), and (**c**) were 11.7%, 22.8%, and 15.2% respectively. The risk factors that exceeded the NCEP thresholds were waist (increased by 24.9%), 18.6%, 30.5%, and 21.6% for low HDLC, 17.4%, 17.4%, and 12.7% for high BP, respectively, for (**o**), (**m**), and (**c**), and 7.1% for high GLUC over the period (see Figure 1). Also the percentages of participants with at least 3 (MS3), 4 (MS4) and 5 (MS5) risk factors beyond the metabolic syndrome NCEP thresholds at Time 2 were almost doubled compared to Time 1. For example, in the FHS-T1 (c) the percentages were 12.2, 5.1, and 1.1, compared to FHS-T2 19.1, 11.6, and 3 percent respectively for MS3, MS4, and MS5. CHD events increased 4% from Time 1 to Time 2. The odds ratios and 95% confidence intervals of having prevalent CHD in the presence of MetS were 2.8 (2.1–3.8), 2.9 (2.2–3.9), and 2.5 (1.9–3.5), respectively, for FHS-T1(**o**), FHS-T1(**m**), FHS-T1(**c**) and 2.3 (1.8–2.9), 4.7 (3.6–6.1), 2.4 (1.9–3.0), respectively, for FHS-T2(**o**), FHS-T2(**m**), and FHS-T2(**c**). A 12–13 fold higher odds were found for T2D in the presence of MetS. T2D percentage had increased from 4.1% in FHS-T1 to 7.3% in FHS-T2.

**Figure 2:**
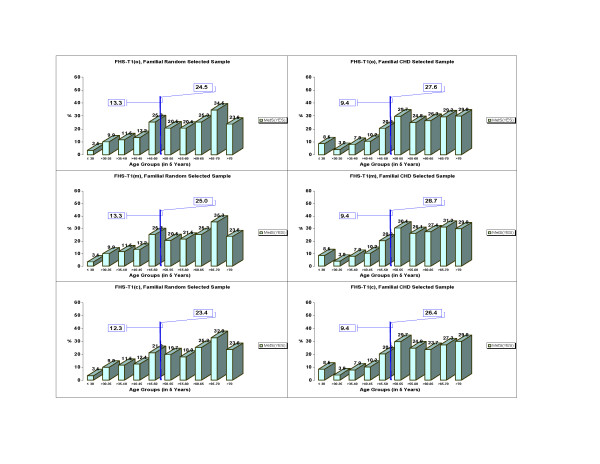
Trends of MetS percentages per age groups in the FHS-Time 1 in a familial random sample and in a familial CHD selected sample. Reported are the corresponding percentages of MetS by 5 years age groups, as well as percentages of ages up to 50, 50 and older for FHS-Time 1, for (**o**), (**m**), and (**c**) methods (see Methods).

**Figure 3:**
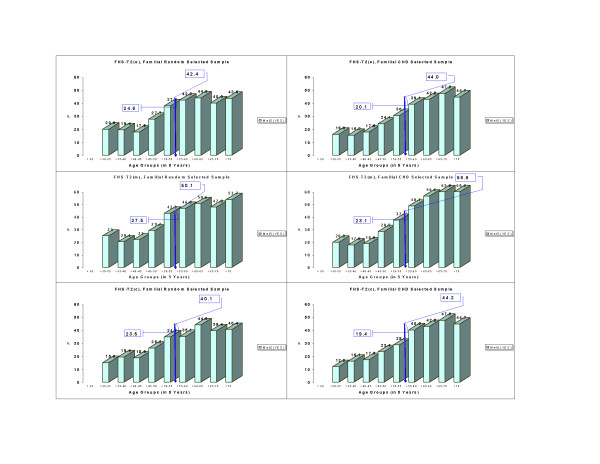
Trends of MetS percentages per age groups in the FHS-Time 2 in a familial random and in a familial CHD selected sample. Reported are the corresponding percentages of MetS by 5 years age groups, as well as percentages of ages up to 55, 55 and older for FHS-Time 2, for (**o**), (**m**), and (**c**) methods (see Methods).

Age also plays an important role in accumulating adverse events that contribute to the MetS expression. The Cochran-Armitage trend tests evidenced significant differences (p < 0.0001) for MetS through 5 age groups (<30, 30–<40, 40–<50, 50–<60, >70) in both FHS-T1 and FHS-T2 (data not shown). Because there was an age shift of the same participants between two time measurements, we compared the prevalence of MetS ages 50 and older for FHS-T1 with those 55 and older for FHS-T2. Such comparison with an age shift of 5 years was performed with the purpose to reduce confounding effect of age. The shift comparison is referred to as 50/55. MetS percentages for participants of age greater or equal to 50/55 increased from FHS-T1 (**o**), (**m**), and (**c**) to FHS-T2, respectively, from 26.1% to 43.3%, from 26.9% to 54.7%, and from 25.0% to 42.2%. Of particular interest were the ratios of MetS percentages for participants 50/55 years or older versus MetS percentages of participants younger than 50/55 years at each time. We expected smaller ratio values when percentages were more similar between the two age groups. The ratios were 2.3 in FHS-T1(**o**), 2.4 in FHS-T1(**m**), 2.3 in FHS-T1(**c**) versus 1.9 in FHS-T2(**o**), 2.1 in FHS-T2(**m**), and 1.9 in FHS-T2(**c**). The above results may point to an increasing secular trend for MetS in the Time 2. These data, furthermore, were separated in familial random and familial CHD selected subsamples (See Methods, and see Figures 2 and 3). For the familial random selected sample, respectively for the three methods (**o**), (**m**), and (**c**), the following increments (11.3, 17.9), (14.2, 25.1), and (11.2, 16.7) in percent (paired for ages younger than 50/55, 50/55 and older) from FHS-T1 to FHS-T2 were identified. For the familial CHD selected sample, in the same order as above, increments of (10.7, 16.4), (13.7, 30.2), and (10.0, 17.8) percent from FHS-T1 to FHS-T2 were documented. There were no statistical differences in the MetS prevalence between males and females in this white matched cohort (results not shown).

## Discussion

This study shows that the percentage of MetS increased substantially over a 7.4 year period in the NHLBI Family Heart Study, and the major contributors to this increase were central obesity, dyslipidemia, and hypertension. Central adiposity (assessed here as waist circumference) appears to be the primary risk factor driving this longitudinal change in MetS prevalence (see Figure 1). Obesity and central obesity have been linked to insulin resistance and hyperinsulinemia and may be the cause of dyslipidemia and hypertension. It should be noted that MetS percentages in ages 50/55 and older were almost 2–3 times higher than in the younger age groups. It can be hypothesized that a life time accumulation of adversities including overnutrition, a sedentary lifestyle, obesity and dyslipidemia, changes in the hormones, untreated hypertension, changes of the functioning of beta cells and other environmental and physiological factors, may trigger a genetic expression of MetS which becomes more prominent with biological maturation [14,17]. The rapid increase in the prevalence of obesity portends a further increase in the prevalence of the metabolic syndrome in the future.

The precision of defining MetS for epidemiological studies is made difficult by the fact that medication treatment can potentially change the level of each risk factor [1,17]. Medication use was exploited by us as an index to correct the level of the actual risk factors in an attempt to estimate the original risk factor levels. In our opinion, most studies of MetS consider the use of medications for changing BP and GLUC risk factors levels as a confirmation that a participant already achieved the NCEP threshold of the specified risk factor. Such considerations are not as common for TG and HDLC since the efficacy of lipid lowering drug classes differ considerably for these traits. In the FHS-T1 there were 0.98% of subjects that used cholesterol lowering drugs and 19.1% that used anti-hypertensive medications. In the FHS-T2 this group broadened to 24.5% that used anti-hyperlipidemics, and to 33.5% that used anti-hypertensive medications. To capture the importance of the lipid lowering medication use in the MetS prevalence estimation, we considered the use of medications for changing BP, lipids, and GLUC risk factor levels as a categorical confirmation that a participant had reached the NCEP threshold. Therefore the difference between MetS percentages estimated by the (**o**) and (**m**) methods within the same time was expected to reflect a change only about the lipids lowering medication use. The difference in the MetS percentages between methods was negligible at the baseline FHS visit [FHS-T1(**m**) and FHS-T1(**o**) (0.4%)], but larger at the follow-up visit [FHS-T2(**m**) and FHS-T2(**o**) (7.9%). This emphasizes the fact that the expansion of the prevalence of lipid lowering medication use can raise the MetS prevalence estimation, and should be considered.

A third approach was performed in an attempt to impute the original levels of treated BP/lipids MetS risk factors (see Methods). This third method resulted in MetS percentages of 18.4% at baseline and 33.6% at follow-up (see Figure 1). We believe that a correction based on the average effects of anti-hypertensives and anti-hyperlipidemics clinical trials on risk factor levels is probably the closest in estimating the MetS prevalence.

For the three methods applied, similar trends were noticed when familial samples were selected random/for CHD (See Methods and see Figures 2 and 3). When lowering the glucose threshold to 100 mg/dl as recommended recently by AHA/NHLBI [17] in comparison to 110 mg/dl recommended by the NCEP MetS definition, the MetS percentages became 22.3%, 22.7%, 21.4% in FHS-T1, and 38.5%, 46.0%, 37.5% in FHS-T2, representing an increment of about 3% of MetS in both times for (**o**), (**m**), and (**c**) methods.

It was apparent, when comparing results of FHS-T1 vs. FHS-T2, that the presence of MetS in the sample was associated with higher odds for prevalent CHD. The odds of having prevalent CHD in the presence of MetS were about 2.5 times higher than in its absence in FHS-T1(**c**) and FHS-T2(**c**). Higher presence of prevalent CHD and T2D when participants were classified with MetS may imply that MetS and/or its contributing risk factors are associated with the development of CHD and T2D. Similar findings were reported in other publications [5,10,21-24].

Our study also has limitations. Although the MetS prevalence in FHS-T2 was noticeably higher than in FHS-T1, in the FHS-T2 sample analyzed we have not accounted for any mortality as result of CHD. Malik et al. examined the impact of MetS on CHD and on the overall mortality in the US adults. They reported a 2.2 hazard ratio for CHD mortality when subjects were classified with MetS [25]. As a result, the estimated prevalence of CHD in FHS-T2 may be conservative. A second possible limitation was related to the recruitment design used in the FHS-T2. Each recruitment center in the study had a goal for the total number of recruited participants and stopped recruiting when they reached the goal. This feature of the recruitment in the FHS-T2 had a potential for bias, especially if the selected sample was biased toward individuals selected for CHD or with familial history of CHD. Although this has been considered to be prevented by each center, we found that 46.6% of the sample was from the original random sample and 53.4% was from the original sample selected for family history of CHD. A third possible limitation is related to the equipment used to measure BP. In the FHS study, blood pressure was measured with two different devices (see Methods). The concern was that the change in equipment may impact the MetS prevalence reported. Although Rose at al [20] informed a skip problem for Dinamap for SBP, Sturrock et al [26] have reported that there is an agreement of sphygmomanometer and Dinamap readings. Also, Kuo et al [27] concluded that the averaged readings of duplicate BP measurements by Dinamap were interchangeable with that by sphygmomanometer. Thus, our method of using the average of the second and third measurements of BP asserts that the two time BP measurements on the same subject are comparable.

Another limitation relates to medication use and MetS definition. In the second method we classified subjects under lipid medication as passing the threshold of MetS for elevated TG and low HDL. This is potentially problematic from a clinical point of view, because not all lipid medications are prescribed for elevated TG or low HDL. Often such medications are prescribed for elevated LDL. From the experience of studying different clinical trials (unpublished data), in this study we assumed that there are correlated medication effects on LDL, HDL, and TG traits. Also for each medication prescribed there are over and under responders. By adjusting everyone on a medication, (regardless of compliance, which in our study was not known), by an average medication efficacy or a percentage there is a potential to over-/under estimate the original imputed HDL, TG, BP, and concurrently the MetS prevalence. Therefore to overcome this limitation, (where data are available), we recommend in similar studies for treated risk factors, their correction per participant with average effects of specific classes of medications matched to specific medication labels [18].

We conclude that when estimating the MetS prevalence it is important to account for the medications use that confounds MetS risk factors. To account categorically for anti-hyperlipidemic medication use is a difficult task that can overestimate MetS prevalence. Our study demonstrated also that MetS is associated with a higher risk for prevalent CHD and T2D. This finding is supported by the scientific literature, which in addition shows that CHD mortality is higher in individuals with MetS than without [25,28]. MetS amplifies with age and is becoming more prevalent over time. Its prevention, by targeting specific MetS risk factors or a combination of them, is a crucial step to interrupt its epidemic.

## Competing interests

The author(s) declare that they have no competing interests.

## Authors' contributions

All authors contributed equally.
